# Supplementary material for: Crohn’s Disease Increases the Mesothelial Properties of Adipocyte Progenitors in the Creeping Fat
Source: Int J Mol Sci. 2021 Apr 20;22(8):4292. doi: 10.3390/ijms22084292 (PMC8074767; doi:10.3390/ijms22084292)
Supplement: Supplementary file 1 [file ijms-22-04292-s001.zip › ijms-1142174 suppl/Supplementary tables 1_2.pdf]

Supplementary Table 1. Human AT and ASCs Microarray Data

Gene ID	Log FC AT	Adjusted p-value AT	Log FC ASCs	Adjusted p-value ASCs	Description
**Mesothelial markers**					
WT1	**5.86**	**3.83E-13**	**1.85**	**1.78E-05**	Wilms tumor 1
MSLN	**4.23**	**7.80E-12**	**1.43**	**4.57E-02**	mesothelin
LRRN4	**3.55**	**1.47E-15**	1.40	1.12E-01	leucine rich repeat neuronal 4
KRT8	**2.15**	**1.45E-10**	1.16	1.07E-01	keratin 8
CDH1	**2.07**	**1.19E-07**	0.92	1.83E-01	cadherin 1
CNN1	**0.76**	**6.18E-03**	0.81	3.73E-01	calponin 1
CDH2	**0.70**	**2.81E-04**	0.55	4.32E-01	cadherin 2
TJP1	**0.27**	**2.44E-02**	0.29	2.90E-01	tight junction protein 1
KRT7	0.09	3.79E-01	0.27	2.08E-01	keratin 7
UPK3A	-0.13	3.64E-01	-0.02	9.82E-01	uroplakin 3A
CALB2	-0.17	3.64E-01	-0.03	8.96E-01	calbindin 2
CA2	-0.30	6.05E-01	-0.17	4.64E-01	carbonic anhydrase 2
THBD	-0.42	1.11E-01	-0.37	8.43E-01	thrombomodulin
SPP1	**-1.12**	**8.88E-03**	**-0.84**	**3.27E-02**	secreted phosphoprotein 1
**Mesenchymal markers**					
CD36	**1.22**	**2.64E-04**	0.33	3.90E-01	CD36 molecule
ENG	0.24	2.51E-01	-0.12	7.91E-01	endoglin
ANPEP	0.08	5.53E-01	**-0.70**	**5.60E-03**	alanyl aminopeptidase, membrane
NT5E	0.05	8.30E-01	**-0.93**	**4.38E-02**	5'-nucleotidase ecto
ITGA4	-0.23	3.01E-01	**-1.0**	**7.66E-04**	integrin subunit alpha 4
THY1	**-0.34**	**9.84E-03**	-1.89	6.58E-02	Thy-1 cell surface antigen
MME	**-1.56**	**1.37E-06**	**-3.83**	**1.26E-05**	membrane metallo-endopeptidase

Microarray analyses revealed that mesothelial markers are upregulated in twenty visceral adipose tissue versus nineteen subcutaneous adipose tissue samples (accession number GSE20950). Microarray analyses revealed that mesothelial markers are upregulated in thirteen visceral AT-derived ASCs versus thirteen subcutaneous AT-derived ASCs samples (accession number GSE37324). Abbreviations: AT, adipose tissue; ASCs, adipose-derived stem cells. Bold text denotes genes with adjusted p-value < 0.05 by limma and adjusted by the Benjamini & Hochberg false discovery rate method.

Supplementary Table 2. Assays contained in TaqMan microfluidic card

Gene	Official symbol	Taqman Assay ID	References
**Mesothelial markers**			
Wilms Tumor 1	WT1	Hs01103751_m1	[1]
mesothelin	MSLN	Hs00245879_m1	[1]
tight Junction Protein 1	TJP1	Hs01551861_m1	[2]
leucine rich repeat neuronal 4	LRNN4	Hs00379905_m1	[3]
uroplakin 3A	UPK3A	Hs00199590_m1	[4]
keratin 7	KRT7	Hs00559840_m1	[3]
keratin 8	KRT8	Hs01595539_g1	[3]
calretinin	CALB2	Hs00242372_m1	[3]
osteopontin	SPP1	Hs00959010_m1	[3]
calponin 1	CNN1	Hs00959434_m1	[3]
thrombomodulin	THBD	Hs00264920_s1	[5]
e-cadherin	CDH1	Hs01023895_m1	[1]
n-cadherin	CDH2	Hs00983056_m1	[6]
carbonic anhydrase 2	CA2	Hs01070108_m1	[1]
**Mesenchymal markers**			
CD73	NT5E	Hs00159686_m1	[7]
CD90	THY1	Hs00174816_m1	[7]
CD105	ENG	Hs00923996_m1	[7]
CD10	MME	Hs00153510_m1	[7, 8]
CD36	CD36	Hs00354519_m1	[7]
CD13	ANPEP	Hs00174265_m1	[8]
CD49d	ITGA4	Hs00168433_m1	[7]
**Housekeeping genes**			
18S rRNA	18S	Hs99999901_s1	
peptidylprolyl isomerase A	PPIA	Hs04194521_s1	

1. Lachaud, C.C., et al., *Use of Mesothelial Cells and Biological Matrices for Tissue Engineering of Simple Epithelium Surrogates.* Front Bioeng Biotechnol, 2015. **3**: p. 117.
2. Lachaud, C.C., et al., *EGF-induced adipose tissue mesothelial cells undergo functional vascular smooth muscle differentiation.* Cell Death Dis, 2014. **5**: p. e1304.
3. Verardo, R., et al., *Specific mesothelial signature marks the heterogeneity of mesenchymal stem cells from high-grade serous ovarian cancer.* Stem Cells, 2014. **32**(11): p. 2998-3011.
4. Chau, Y.Y., et al., *Visceral and subcutaneous fat have different origins and evidence supports a mesothelial source.* Nat Cell Biol, 2014. **16**(4): p. 367-75.

5. Verhagen, H.J., et al., *Thrombomodulin activity on mesothelial cells: perspectives for mesothelial cells as an alternative for endothelial cells for cell seeding on vascular grafts.* Br J Haematol, 1996. **95**(3): p. 542-9.
6. Mutsaers, S.E., et al., *Mesothelial cells in tissue repair and fibrosis.* Front Pharmacol, 2015. **6**: p. 113.
7. Bourin, P., et al., *Stromal cells from the adipose tissue-derived stromal vascular fraction and culture expanded adipose tissue-derived stromal/stem cells: a joint statement of the International Federation for Adipose Therapeutics and Science (IFATS) and the International Society for Cellular Therapy (ISCT).* Cytotherapy, 2013. **15**(6): p. 641-8.
8. Bajek, A., et al., *Adipose-Derived Stem Cells as a Tool in Cell-Based Therapies.* Arch Immunol Ther Exp (Warsz), 2016. **64**(6): p. 443-454.
