# Supplementary material for: Crohn’s Disease Increases the Mesothelial Properties of Adipocyte Progenitors in the Creeping Fat
Source: Int J Mol Sci. 2021 Apr 20;22(8):4292. doi: 10.3390/ijms22084292 (PMC8074767; doi:10.3390/ijms22084292)

Supplementary Figure 1

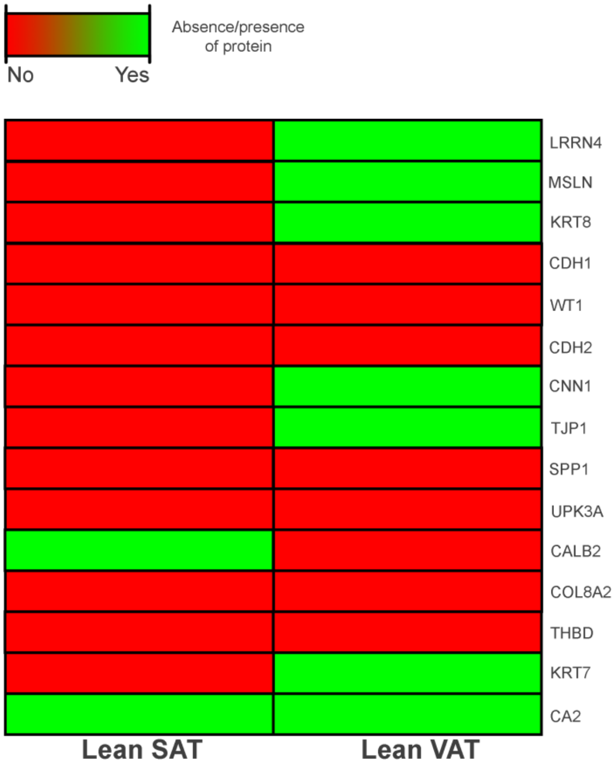

Supplementary Figure 2

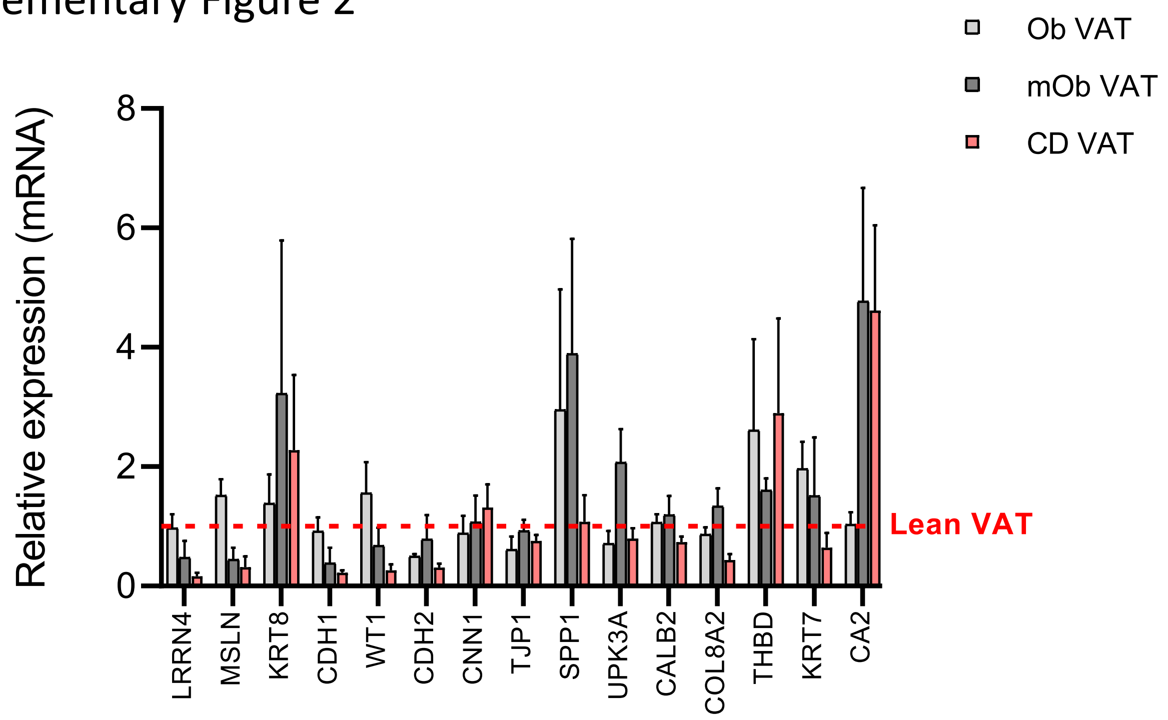

Supplementary Figure 3

A

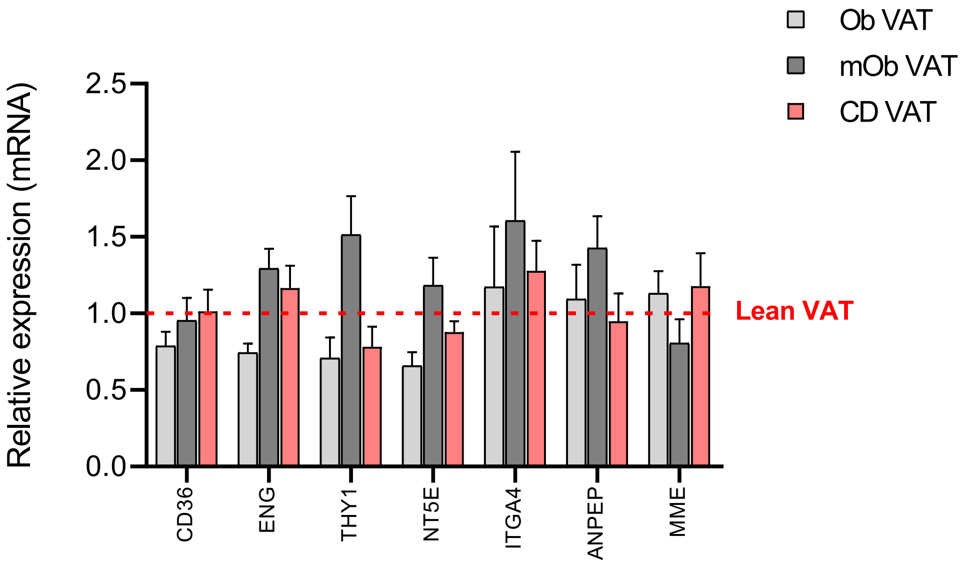

B

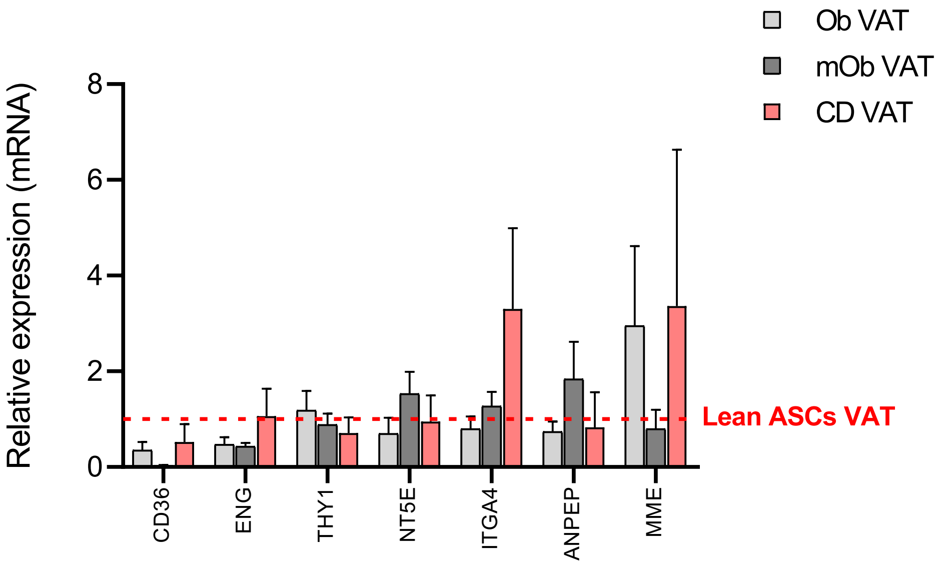

Supplementary Figure 4

A

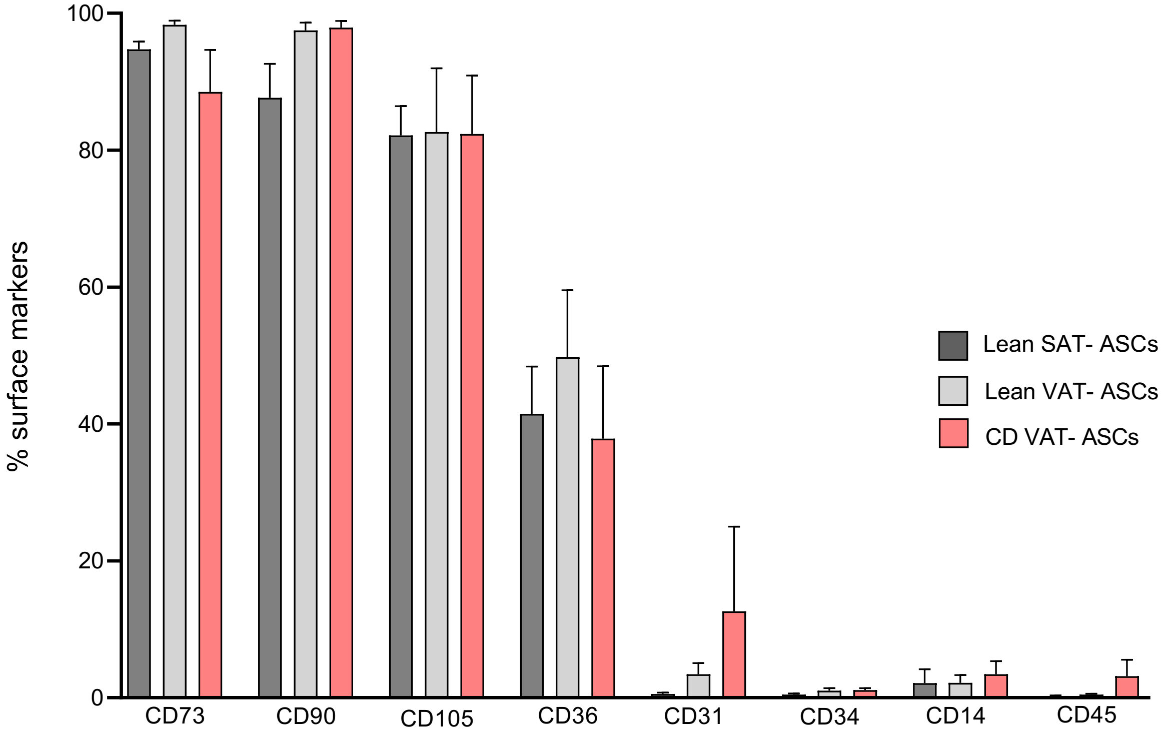

B

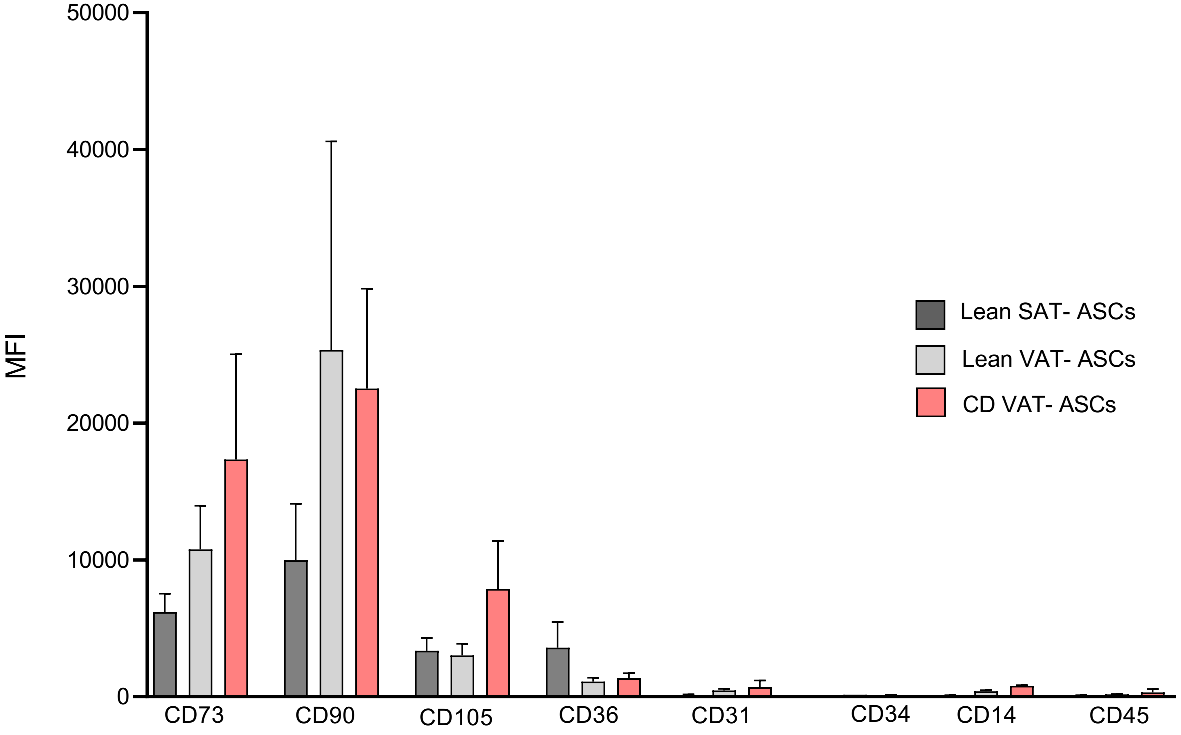

Supplement: Supplementary file 1 [file ijms-22-04292-s001.zip › ijms-1142174 suppl/Supplementary_Figures_Madeira.pdf]
